# Supplementary material for: Wolf spider burrows from a modern saline sandflat in central Argentina: morphology, taphonomy and clues for recognition of fossil examples
Source: PeerJ. 2018 Jun 29;6:e5054. doi: 10.7717/peerj.5054 (PMC6027663; doi:10.7717/peerj.5054)
Supplement: Supplemental Information 1 — Length = 131 mm; Neck Length = 8 mm; Minimum Diameter = 15 mm; Maximum Diameter = 22 mm; Angle = 84º. Dweller captured (Pavocosa sp GHUNLPam-4770). 3D model credit: Fatima Mendoza-Belmontes. [file peerj-06-5054-s001.pdf]

Mendoza-Belmontes et al. (2018). Wolf spider burrows from a modern saline sandflat in central Argentina: morphology, taphonomy and clues for recognition of fossil examples. Journal PeerJ.

Additional File: Interactive 3D PDF

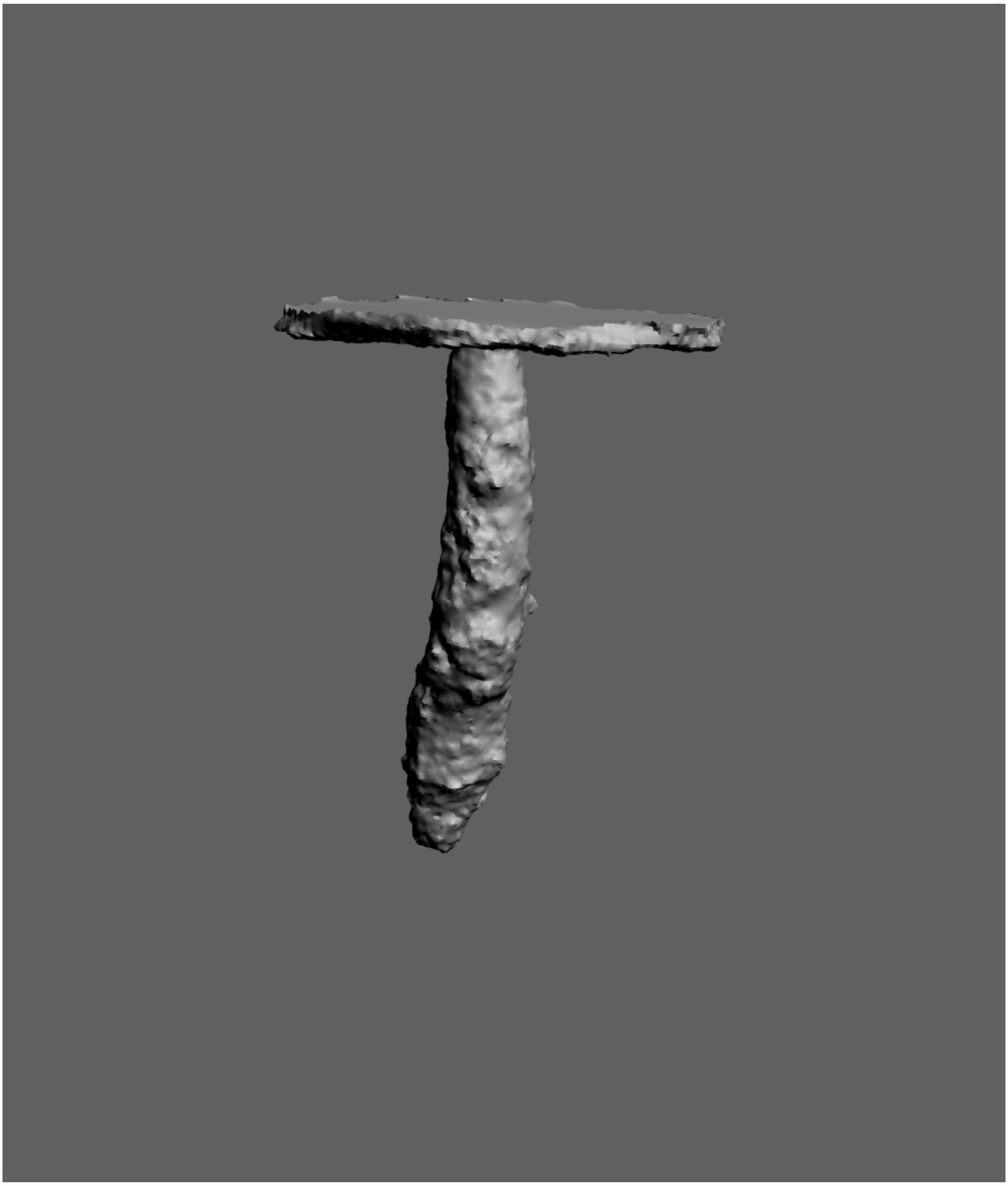

Figure 1. Cast GHUNLPam-4771. Length= 131 mm; Neck Length= 8 mm; Minimum Diameter = 15 mm; Maximum Diameter= 22 mm; Angle= 84º. Dweller captured (*Pavocosa* sp GHUNLPam-4770).
