# Supplementary material for: Wolf spider burrows from a modern saline sandflat in central Argentina: morphology, taphonomy and clues for recognition of fossil examples
Source: PeerJ. 2018 Jun 29;6:e5054. doi: 10.7717/peerj.5054 (PMC6027663; doi:10.7717/peerj.5054)
Supplement: Supplemental Information 3 — Length = 130 mm; Neck Length = 6 mm; Minimum Diameter = 16 mm; Maximum Diameter = 27 mm; Angle = 75º. Dweller captured (Pavocosa sp GHUNLPam-4770). Eggs sac found on the bottom. 3D model credit: Fatima Mendoza-Belmontes. [file peerj-06-5054-s003.pdf]

Mendoza-Belmontes et al. (2018). Wolf spider burrows from a modern saline sandflat in central Argentina: morphology, taphonomy and clues for recognition of fossil examples. Journal PeerJ.

Additional File: Interactive 3D PDF

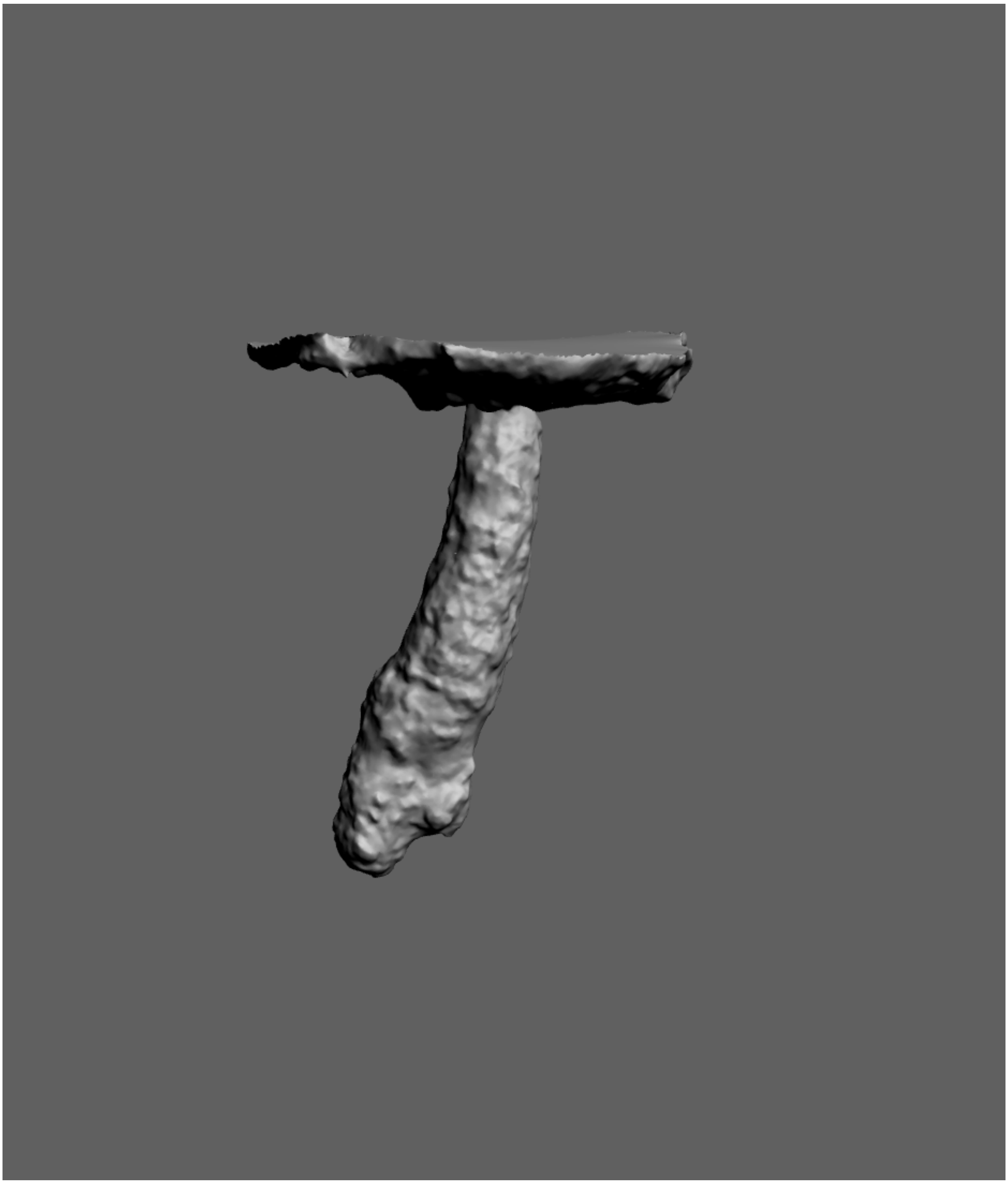

Figure 3. Cast GHUNLPam-4773. Length= 130 mm; Neck Length= 6 mm; Minimum Diameter= 16 mm; Maximum Diameter= 27 mm; Angle= 75º. Dweller captured (*Pavocosa* sp GHUNLPam-4770). Eggs sac found on the bottom.
