# Supplementary material for: Wolf spider burrows from a modern saline sandflat in central Argentina: morphology, taphonomy and clues for recognition of fossil examples
Source: PeerJ. 2018 Jun 29;6:e5054. doi: 10.7717/peerj.5054 (PMC6027663; doi:10.7717/peerj.5054)
Supplement: Supplemental Information 4 — Length = 106 mm; Neck Length = 6 mm; Minimum Diameter = 13 mm; Maximum Diameter = 23 mm; Angle = 72º. Sac of eggs found on the bottom. 3D model credit: Fatima Mendoza-Belmontes. [file peerj-06-5054-s004.pdf]

Mendoza-Belmontes et al. (2018). Wolf spider burrows from a modern saline sandflat in central Argentina: morphology, taphonomy and clues for recognition of fossil examples. Journal PeerJ.

Additional File: Interactive 3D PDF

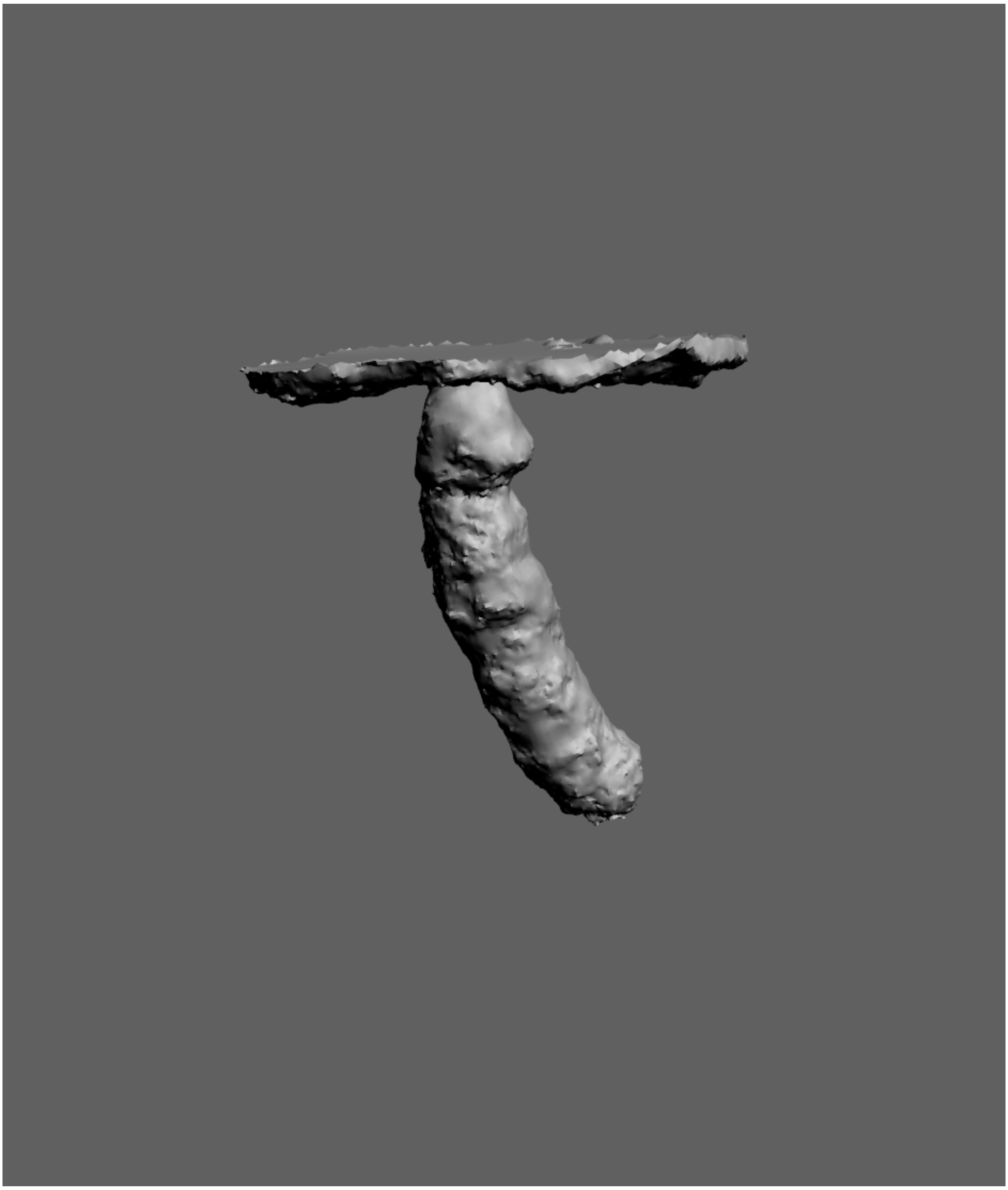

Figure 5. Cast GHUNLPam-4774. Length= 106 mm; Neck Length= 6 mm; Minimum Diameter= 13 mm; Maximum Diameter= 23 mm; Angle= 72º. Sac of eggs found on the bottom.
