# Supplementary material for: Wolf spider burrows from a modern saline sandflat in central Argentina: morphology, taphonomy and clues for recognition of fossil examples
Source: PeerJ. 2018 Jun 29;6:e5054. doi: 10.7717/peerj.5054 (PMC6027663; doi:10.7717/peerj.5054)
Supplement: Supplemental Information 5 — Length = 118 mm; Neck Length = 7 mm; Minimum Diameter = 13 mm; Maximum Diameter = 25 mm; Angle = 85º. 3D model credit: Fatima Mendoza-Belmontes. [file peerj-06-5054-s005.pdf]

Mendoza-Belmontes et al. (2018). Wolf spider burrows from a modern saline sandflat in central Argentina: morphology, taphonomy and clues for recognition of fossil examples. Journal PeerJ.

Additional File: Interactive 3D PDF

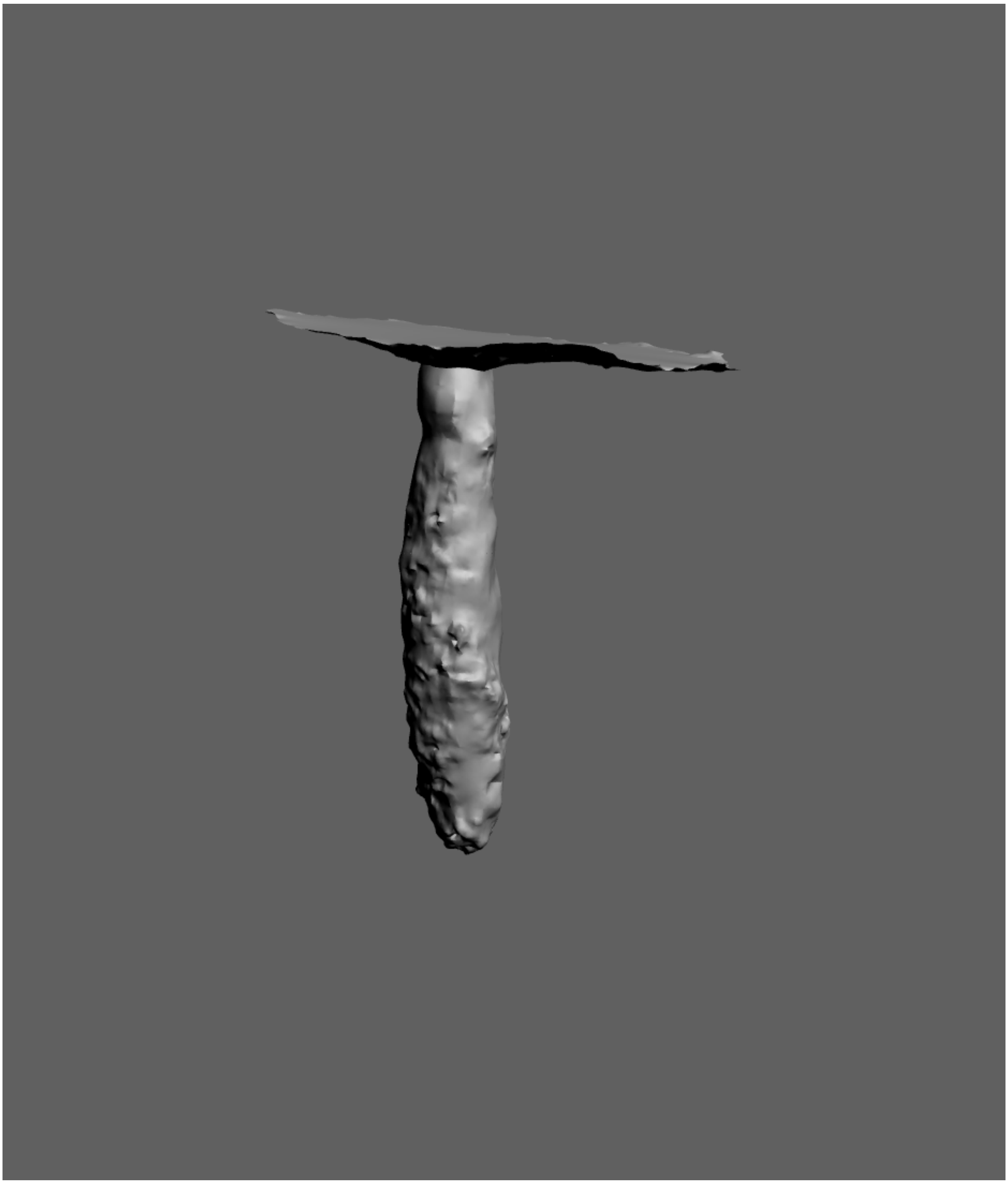

Figure 5. Cast GHUNLPam-4775. Length = 118 mm; Neck Length= 7 mm; Minimum Diameter= 13 mm; Maximum Diameter= 25 mm; Angle= 85º.
