# Supplementary material for: Wolf spider burrows from a modern saline sandflat in central Argentina: morphology, taphonomy and clues for recognition of fossil examples
Source: PeerJ. 2018 Jun 29;6:e5054. doi: 10.7717/peerj.5054 (PMC6027663; doi:10.7717/peerj.5054)
Supplement: Supplemental Information 6 — Length = 111 mm; Minimum Diameter = 15 mm; Maximum Diameter = 15 mm; Angle = 86º; ”Umbrella” Structure: Diameter = 47 × 59 mm. 3D model credit: Fatima Mendoza-Belmontes. [file peerj-06-5054-s006.pdf]

Mendoza-Belmontes et al. (2018). Wolf spider burrows from a modern saline sandflat in central Argentina: morphology, taphonomy and clues for recognition of fossil examples. Journal PeerJ.

Additional File: Interactive 3D PDF

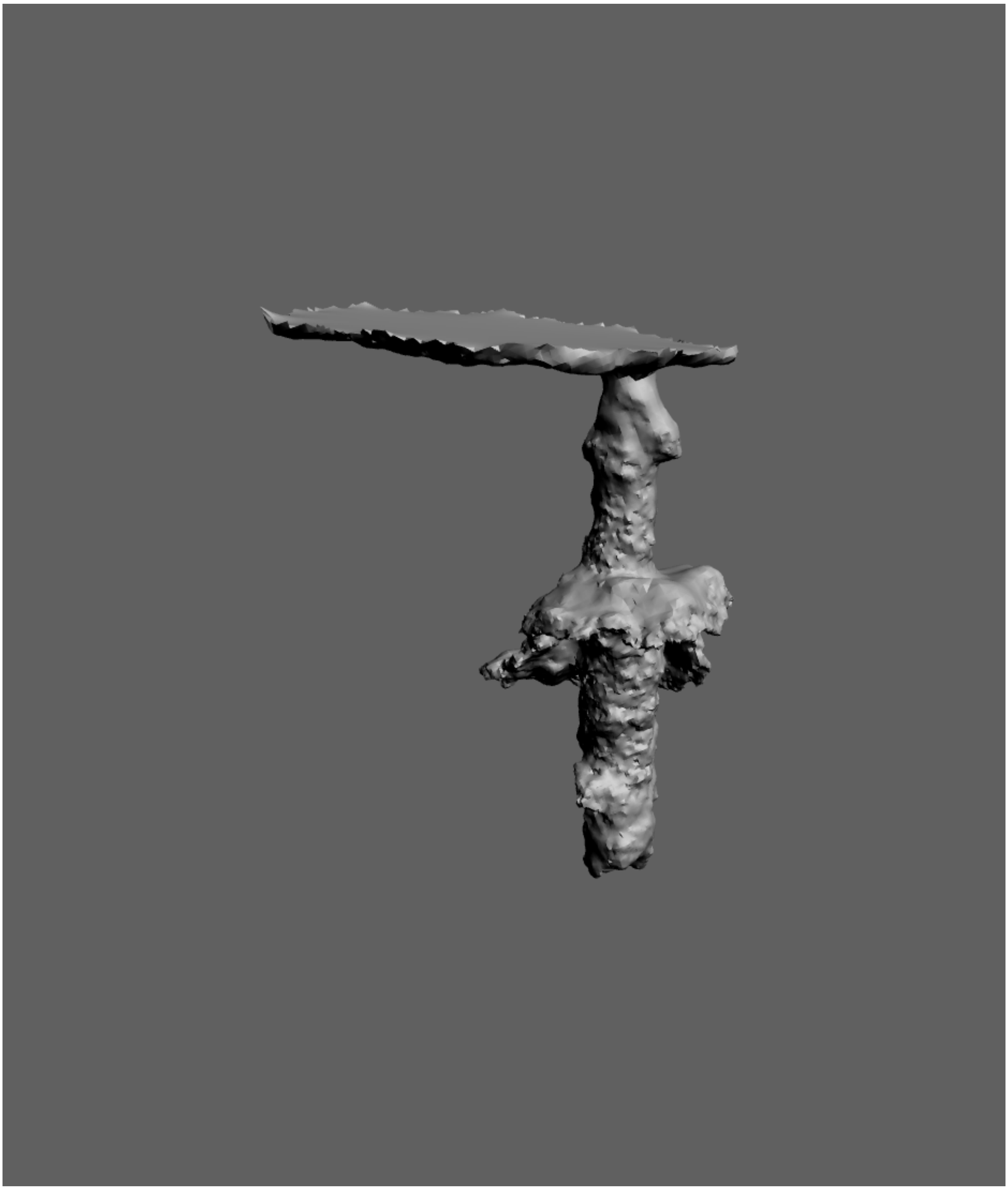

Figure 6. Cast GHUNLPam-4776. Length= 111 mm; Minimum Diameter= 15 mm; Maximum Diameter= 15 mm; Angle= 86º; "Umbrella" Structure Diameter= 47 x 59 mm.
