# Supplementary material for: Wolf spider burrows from a modern saline sandflat in central Argentina: morphology, taphonomy and clues for recognition of fossil examples
Source: PeerJ. 2018 Jun 29;6:e5054. doi: 10.7717/peerj.5054 (PMC6027663; doi:10.7717/peerj.5054)
Supplement: Supplemental Information 9 — Cast GHUNLPam-4778. Length = 116 mm; Minimum Diameter = 15 mm; Maximum Diameter = 22 mm; Angle = 87º. Smaller burrows with around 33 mm length and 8 mm of diameter. 3D model credit: Fatima Mendoza-Belmontes. [file peerj-06-5054-s009.pdf]

Mendoza-Belmontes et al. (2018). Wolf spider burrows from a modern saline sandflat in central Argentina: morphology, taphonomy and clues for recognition of fossil examples. Journal PeerJ.

Additional File: Interactive 3D PDF

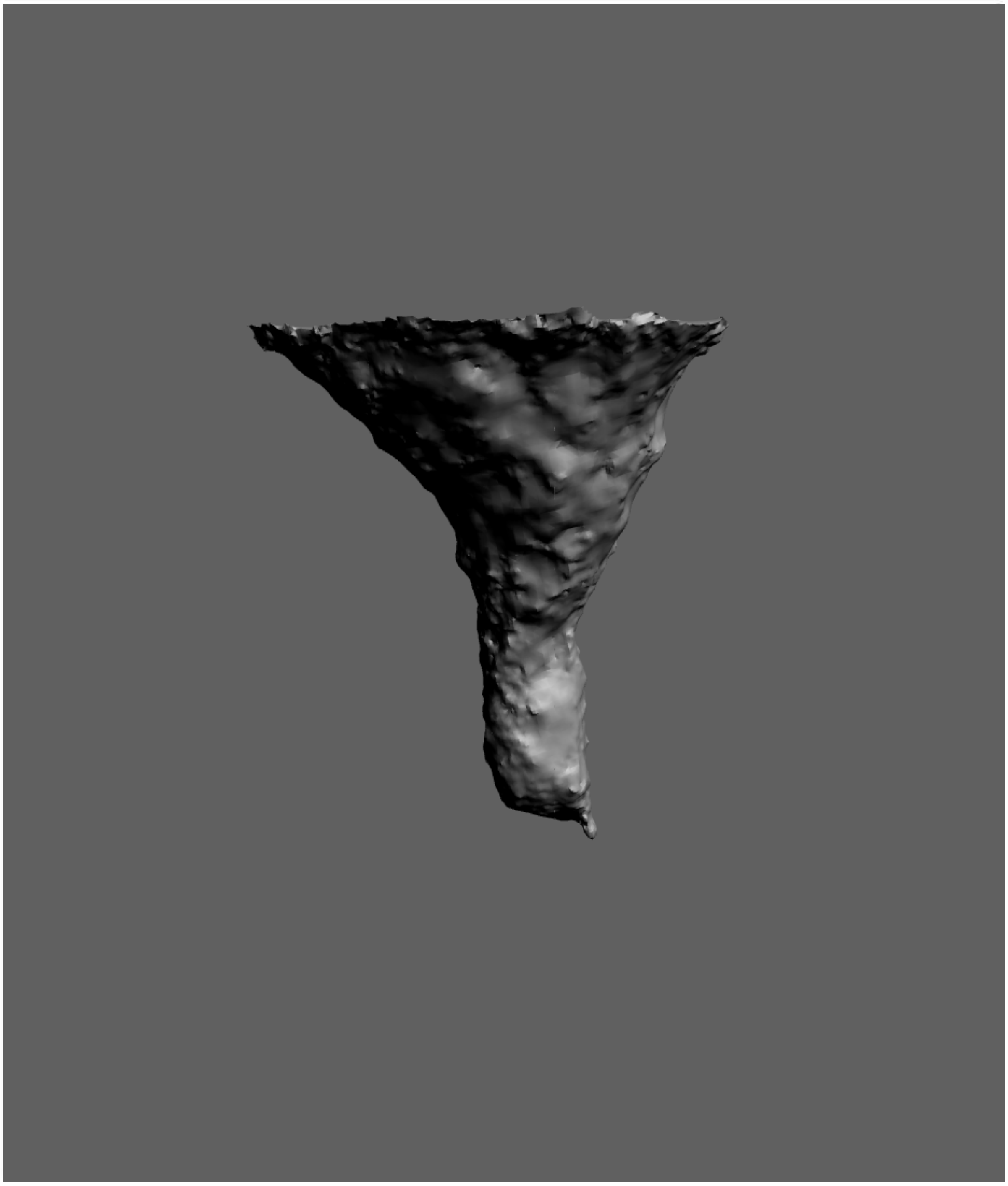

Figure 9. Cast GHUNLPam-4779. Length= 130 mm; Minimum Diameter= 12 mm; Maximum Diameter= 100 mm; Angle= 85º.
